# New entity of microsecretory adenocarcinoma of salivary glands: first case with recurrence and metastases — proof of malignancy

**DOI:** 10.1007/s00428-022-03374-9

**Published:** 2022-07-02

**Authors:** Philipp Jurmeister, Christian Haas, Wolfgang Eisterer, Hermann Rogatsch, Stephan Ihrler

**Affiliations:** 1 Institute of Pathology, Ludwig Maximilians University, Thalkirchner Straße 36, 80337 Munchen Munich, Germany; 2 German Cancer Consortium (DKTK), Partner Site Munich, and German Cancer Research Center (DKFZ), Heidelberg, Germany; 3 DERMPATH München, Munich, Germany; 4 Department for Medical Oncology, Klinikum Klagenfurt a.Ws., Klagenfurt, Austria; 5 Institute of Pathology, Klinikum Klagenfurt a. Ws., Klagenfurt, Austria

**Keywords:** Microsecretory adenocarcinoma, Salivary gland tumors, Head and neck cancer, Metastases, Local recurrence

## Abstract

Microsecretory adenocarcinoma (MSA) of the salivary glands is a recently described entity. Due to lack of reported metastases, in 30 cases described until now, the designation as low-grade cancer was so far solely based on demonstration of local tumor invasion and in a single case with perineural invasion. We herein describe the first documented case with local recurrence and hematogenous metastases.

## Case report

In 2009, a 62-year-old man presented with an intraoral mass at the right cheek. Histology revealed a moderately cell-rich tumor with predominant tubular and microcystic growth pattern, without nuclear atypia and with low proliferative activity (Ki67 2%). The tumor was classified as low-grade mucoepidermoid carcinoma. Despite positive margins, a final resection was not performed.

**Fig. 1:**
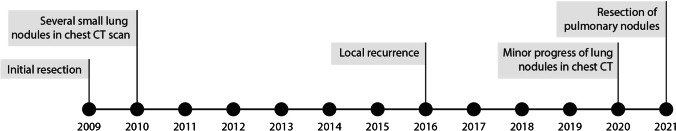
Timeline with an overview of the most important clinical and radiological findings

**Fig. 2:**
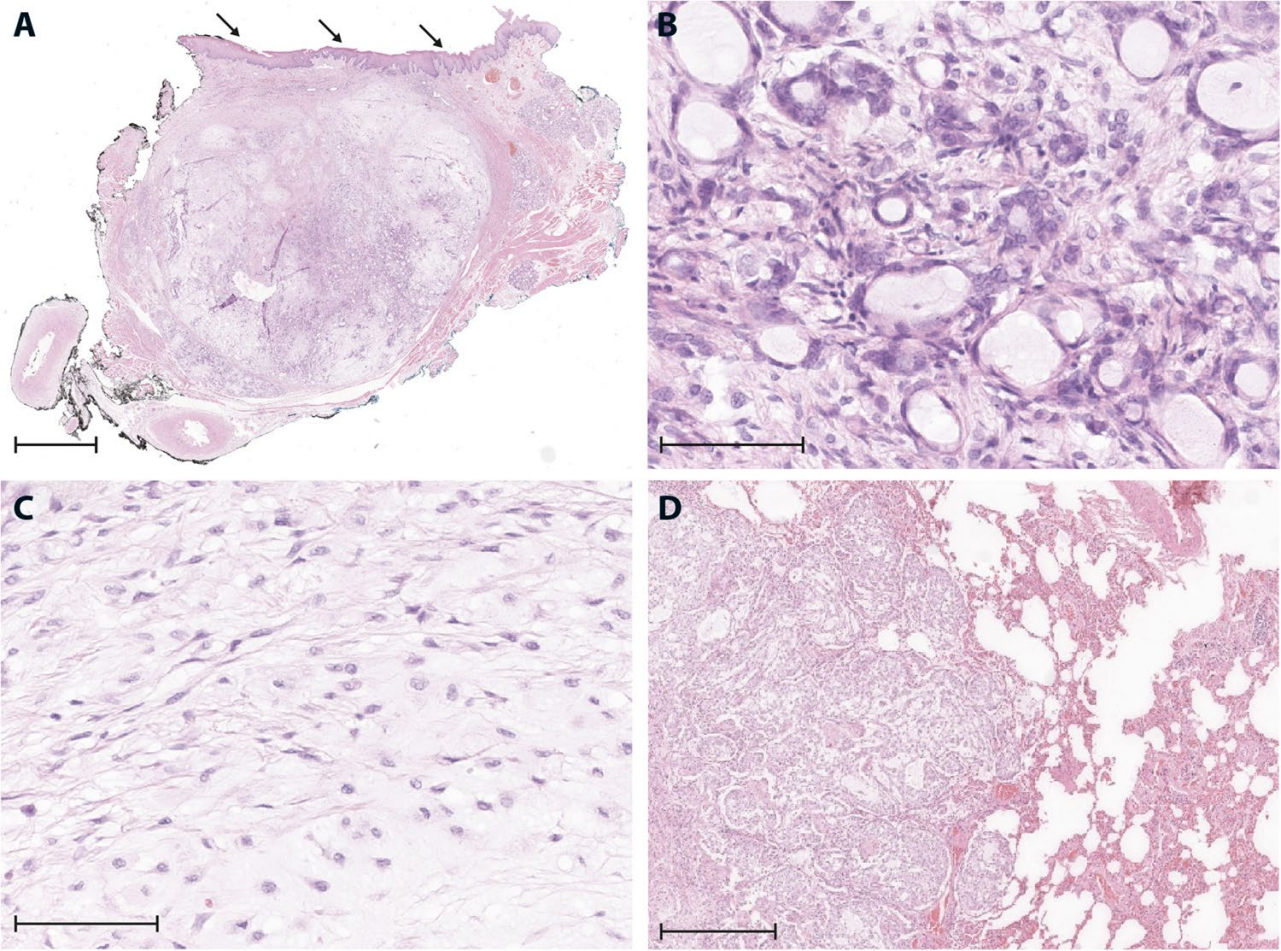
**A** Overview (1.4 × ; scale bar: 500 µm) of the resection specimen, showing a largely well-circumscribed, focally infiltrative, and variably cellular, submucosal tumor (arrows: mucosa). **B** High-power magnification (40 ×) from cell-rich area with monomorphic, unilayered tubules and microcysts with bland cytological features. **C** Cell-poor area with monomorphic spindle cells, representing fibroblastic cells or modified myoepithelial cells (40 × ; scale bar: 40 µm). **D** Metastatic nodule in the lung with cell-rich tumor component (10 × ; scale bar: 100 µm)

Local recurrence manifested 7 years later, measuring 2 cm (Fig. 1). The resection specimen (Fig. 2A–C) contained large, cell-poor areas with spindle cells and myxoid stroma, as well as smaller areas with higher cellularity and monomorphic, tubular to microcystic structures with unilayered cuboidal epithelium and co-expression of CK14, CK18, p63, and SOX10. The small lumina contained PAS-positive mucous substance. The diagnosis of pleomorphic adenoma (recurrence) was made.

In a thoracic computed tomography (CT), performed 1 year after the initial tumor manifestation, several small intrapulmonary nodules of unclear etiology were identified. A control CT in 2020 revealed minor growth of the pulmonary nodules (up to 1.2 cm), and a diagnostic resection of two nodules was performed. Histological evaluation revealed identical tumor tissue as in the primary and recurrent tumor in the cheek, identifying pulmonary metastases of an obviously low-grade malignant salivary tumor (Fig. 2D).

Material of the primary and recurrent tumor, as well as of the pulmonary metastases, was submitted for expert reference (S.I.). Based on publications of a new entity, available only from 2019 on [1], the diagnosis of MSA with local recurrence and pulmonary metastases was retrospectively rendered, based on typical histology and immunohistology, and on proof of a tumor-specific *MEF2C::SS18* gene fusion in reverse transcription polymerase chain reaction (RT-PCR) and fluorescence in situ hybridization (FISH) analysis of both the primary tumor and lung metastasis.

## Discussion

As many other entities of low-grade salivary carcinomas, MSA is characterized by major absence of cytological atypia and by low proliferative activity (low mitotic count and Ki67 usually < 5% according to the author’s own experience on a small case series) [1]. It exhibits uniform microcystic tubules with unilayered, cuboideal epithelium and PAS-positive luminal secretion and a consistent, but not specific immunophenotype (co-expression of CK14, CK18, S100, SOX10, p63 and negativity for p40, mammaglobin, and calponin) [1–3]. On molecular basis, MSA is characterized by an obviously obligate and specific *MEF2C::SS18* gene fusion, which can be detected using RT-PCR or FISH [1, 4, 5].

The follow-up data on 30 patients, reported in the literature, did not so far show any case with recurrence or metastatic disease [1, 3, 6]. Hence, in the absence of cellular atypia or increased proliferation, focal infiltration into surrounding glandular or soft tissue and perineural invasion in a single case have been the only clues to classify this new tumor entity as malignant [1, 3].

The present case with the first documented local recurrence and (pulmonary) metastases proves without doubt that the assumption of the first descriptor of a malignant character of this tumor type was correct [1]. The fact that this is the first description of a recurrent and metastasized MSA within 31 reported cases, and that the pulmonary metastases showed only minor progression within 10 years, strongly supports that this new salivary carcinoma entity is low grade, as are many other salivary carcinoma types, especially in the minor glands [7].

Lack of cellular atypia and of increased proliferation in MSA and in other carcinoma types of the minor salivary glands complicates histological classification and delimitation from benign tumors (e.g., pleomorphic adenoma), especially if only limited material from biopsies is available [7]. The presented case is in addition special, as the dominating, cell-poor areas with fibro-myxoid stroma are strongly reminiscent of pleomorphic adenoma (Fig. 2C). An important clue for MSA is that the characteristic monomorphic tubules/microcysts are unilayered (Fig. 2B), while the tubules in pleomorphic adenoma are bilayered. Furthermore, MSA can be distinguished from secretory carcinoma by the presence of characteristic monomorphous tubules, immunohistochemistry (positivity for p63 and negativity for mammaglobin), and different gene fusions [8].

In summary, with the first documented case of late recurrence and metastases, we provide unquestionable evidence for the previous assumption, that MSA represents a malignant entity with low-grade character.

## References

[1] Bishop JA, Weinreb I, Swanson D, Westra WH, Qureshi HS, Sciubba J, u. a. (2019) Microsecretory adenocarcinoma. Am J Surg Pathol 43:1023–32 10.1097/PAS.0000000000001273 31094920

[2] Rooper LM (2021). Emerging entities in salivary pathology a practical review of sclerosing microcystic adenocarcinoma, microsecretory adenocarcinoma, and secretory myoepithelial carcinoma. Surg Pathol Clin.

[3] Bishop JA, Sajed DP, Weinreb I, Dickson BC, Bilodeau EA, Agaimy A, u. a. (2021) Microsecretory adenocarcinoma of salivary glands: an expanded series of 24 cases. Head Neck Pathol 15:1192–201 10.1007/s12105-021-01331-7 PMC8633253 33982215

[4] Bishop JA, Koduru P, Veremis BM, Oliai BR, Weinreb I, Rooper LM, u. a. (2021) SS18 Break-apart fluorescence in situ hybridization is a practical and effective method for diagnosing microsecretory adenocarcinoma of salivary glands. Head Neck Pathol 15:723–6 10.1007/s12105-020-01280-7 PMC8385014 33394377

[5] Skálová A, Hyrcza MD, Vaneček T, Baněčková M, Leivo I (2022). Fusion-positive salivary gland carcinomas. Genes Chromosomes Cancer.

[6] Kawakami F, Nagao T, Honda Y, Sakata J, Yoshida R, Nakayama H, u. a. (2020) Microsecretory adenocarcinoma of the hard palate: a case report of a recently described entity. Pathol Int 70:781–5 10.1111/pin.12987 32687666

[7] Ihrler S, Agaimy A, Guntinas-Lichius O, Haas CJ, Mollenhauer M, Sandison A, u. a. (2021) Why is the histomorphological diagnosis of tumours of minor salivary glands much more difficult? Histopathology 79:779–90 10.1111/his.14421 34042205

[8] Skálová A, Vanecek T, Sima R, Laco J, Weinreb I, Perez-Ordonez B, u. a. (2010) Mammary analogue secretory carcinoma of salivary glands, containing the ETV6-NTRK3 Fusion Gene: a hitherto undescribed salivary gland tumor entity. Am J Surg Pathol 34:599–608 10.1097/PAS.0b013e3181d9efcc 20410810

